# Measurements of Salivary Alpha Amylase and Salivary Cortisol in Hominoid Primates Reveal Within-Species Consistency and Between-Species Differences

**DOI:** 10.1371/journal.pone.0060773

**Published:** 2013-04-17

**Authors:** Verena Behringer, Claudia Borchers, Tobias Deschner, Erich Möstl, Dieter Selzer, Gottfried Hohmann

**Affiliations:** 1 Department of Primatology, Max Planck Institute for Evolutionary Anthropology, Leipzig, Germany; 2 Department of Biomedical Sciences / Biochemistry, University of Veterinary Medicine, Vienna, Austria; 3 Working Group for Wildlife Biology, Justus-Liebig-University Gießen, Gießen, Germany; Federal University of Parana (UFPR) – Campus Palotina, Brazil

## Abstract

Salivary alpha amylase (sAA) is the most abundant enzyme in saliva. Studies in humans found variation in enzymatic activity of sAA across populations that could be linked to the copy number of loci for salivary amylase (AMY1), which was seen as an adaptive response to the intake of dietary starch. In addition to diet dependent variation, differences in sAA activity have been related to social stress. In a previous study, we found evidence for stress-induced variation in sAA activity in the bonobos, a hominoid primate that is closely related to humans. In this study, we explored patterns of variation in sAA activity in bonobos and three other hominoid primates, chimpanzee, gorilla, and orangutan to (a) examine if within-species differences in sAA activity found in bonobos are characteristic for hominoids and (b) assess the extent of variation in sAA activity between different species. The results revealed species-differences in sAA activity with gorillas and orangutans having higher basal sAA activity when compared to *Pan*. To assess the impact of stress, sAA values were related to cortisol levels measured in the same saliva samples. Gorillas and orangutans had low salivary cortisol concentrations and the highest cortisol concentration was found in samples from male bonobos, the group that also showed the highest sAA activity. Considering published information, the differences in sAA activity correspond with differences in AMY1 copy numbers and match with general features of natural diet. Studies on sAA activity have the potential to complement molecular studies and may contribute to research on feeding ecology and nutrition.

## Introduction

Salivary alpha amylase (sAA) is the most abundant enzyme in saliva [1] and it is primarily produced after neurotransmitter stimulation by the acinar cells in the salivary glands [2]. The amount of sAA excretion is independent of salivary flow rate [3] and the major function of sAA is to assist transformation of dietary carbohydrates by hydrolyzing α-1,4 linkages of starch into maltose, maltrotriose and larger oligosaccharides. In addition to this function, sAA plays a role in a variety of processes related to oral health [4], [5].

While sAA activity has been shown for mammalian taxa with diverse dietary habits such as carnivores [6], [7], rodents [7], [8] and nonhuman primates [9], [10], [11], [12], it is apparently missing in all ruminants investigated so far [6], [7]. Within the primate order New World monkeys show no quantifiable amount of sAA [8], [13] whereas some Old World monkeys such as baboons and macaques have high concentrations of sAA (e.g. [10], [14], [15]). Likewise, sAA activity has been detected in all species of hominoidae [11], [12], [16], [17].

Data from humans reveal considerable variation in enzymatic activity of sAA across populations [18], [19] ranging from high levels in agricultural societies to low levels in circum-arctic hunter-gatherers populations [16]. The patterns of between-population differences have been linked to the number of AMY1 copies (AMY 1 is the loci for salivary amylase) which is seen as an adaptive response to the intake of dietary starch [16]. The same has been reported for non-human primates [17]. In many species, dietary patterns are relatively consistent over time and variation between populations in terms of sAA activity is expected to be stable. In addition to diet dependent variation, differences in sAA activity have been detected in response to social and psychological stress [20], [21] and there is evidence for considerable inter-individual variation in sAA activity [22], [23].

Studies on humans have emphasized the role of dietary starch as a driving force for the AMY1 polymorphism. Compared to humans, hominoid primates have much lower numbers of AMY1 copies and it has been argued that this may be related to low intake of dietary starch by hominoids (e.g. [16]). The species of hominoid primates differ in terms of AMY1 copy numbers but the range of variation is modest when compared with humans. Wilson et al. [24] reported higher relative copy numbers of AMY1 in gorillas compared to chimpanzees. Furthermore, chimpanzees have only two, and bonobos have four AMY1 diploid copy numbers [16]. As coding sequences for AMY1 in bonobos are disrupted, it has been assumed that their function may be reduced or not functional [16] and the two *Pan* species may be more similar in terms of sAA activity as indicated by the difference in AMY1 copy numbers. To our knowledge there are no published data on the AMY1 copy number of orangutans, the Asian hominoid primate species. One conference abstract hints that “… humans and orangutans (have) increased AMY1 copy numbers and …. higher salivary amylase…” [25], rendering correlations between AMY1 copy number and sAA activity in orangutans speculative. A recent study by Behringer et al. [12] found that bonobos have sAA activity and the sAA activity of bonobos showed significant sex differences with males ranging higher than females. There is no indication that male and female bonobos differ in terms of AMY1 copy numbers or in terms of their diet which could explain the observed sex differences. A possible explanation for this finding is that sex differences in sAA activity are related to stress. Based on the results obtained from the bonobo data set, the only one that was sufficiently large to facilitate such analyses, the authors found evidence for a relationship between sAA activities and stress [12]. There is evidence that variation in glucocorticoid levels, another physiological marker for stress, can be explained by species specific patterns of stress exposure and coping mechanisms [26]. By inference, one may speculate that in hominoid primates within species variation in sAA activity may reflect exposure to stress rather than differences in diet. Traditionally, monitoring short-term stress response non-invasively in great apes has been performed by measuring salivary cortisol levels (e.g. chimpanzee: [27], bonobo: [28], orangutan: [29], western lowland gorilla: [30]).

Here we present a data set on measures of sAA activity in four species of great apes. The first aim is to examine if within-species differences in sAA activity found in bonobos are characteristic for other hominoids. The second aim is to assess the extent of variation in sAA activity between different species of great apes. If – as in humans - sAA activity correlates positively with AMY1 copy numbers, we expect to find two clusters in the data from apes, that is, consistently higher sAA activity in gorillas and orangutans and low levels in the two *Pan* species. In case sAA activity reflects a response to stress, we expect symmetry in terms of cortisol levels and sAA activity. Accordingly, we measured sAA and salivary cortisol simultaneously.

## Methods

### Ethics Statement

The protocol of sample collection was approved by the scientific authorities of the following zoos: Zoo Berlin (Dr. Andrè Schüle), Zoo Frankfurt (Dr. Christian R. Schmidt and Dr. Thomas Wilms), Zoo Heidelberg (Dr. Sandra Reichler), Zoo Krefeld (Dipl. Biol. Cornelia Bernhardt), Zoo Leipzig (Dr. Andreas Bernhard), Zoo Munich (Dipl. - Biol. Beatrix Köhler and Dipl.-Biol. Carsten Zehrer), and Zoo Wuppertal (Dipl. - Biol. André Stadler).

### Subjects

Multiple saliva samples (N_samples_ = 669) were collected from December 2006 to October 2011 from individual bonobos (N_individuals_ = 21, age range: 1–49 years of age, table S1), chimpanzees (N = 24, age range: 8–50 years of age, table S1), orangutans (N = 24, age range: 2–48 years of age, table S1) and western lowland gorillas (N = 13, age range: 1–51 years of age, table S1) kept at eight zoos (table 1) with a volume range from 10 µl to 1350 µl. Most samples (N = 659) contained sufficient amounts of saliva to measure salivary cortisol as well as sAA. In 10 samples the volume of saliva was low allowing only measurement of sAA. All apes lived in social groups at all times, except one male orangutan which was temporary isolated from the group. For all apes indoor and outdoor enclosures were provided. Apes received a mix of fruits and vegetables several times per day and had *ad libitum* access to fresh water. Overall, the diet consumed by the different species was rather similar within each zoo facility.

**Table 1:** Species, location, sex and number of saliva samples used for measuring amylase and cortisol.

species	location	male	female	salivaryamylase (N)	salivary cortisol (N)
Bonobo	Berlin	3	0	33	29
Bonobo	Frankfurt	4	10	144	142
Bonobo	Leipzig	3	0	18	18
Bonobo	Wuppertal	1	0	4	4
Chimpanzee	Berlin	2	2	34	34
Chimpanzee	Leipzig	1	10	110	110
Chimpanzee	Munich	3	2	21	19
Chimpanzee	Nordhorn	1	3	19	19
Borneo orangutan	Berlin	1	0	5	5
Borneo orangutan	Krefeld	2	2	19	19
Sumatran orangutan	Berlin	3	4	67	67
Sumatran orangutan	Frankfurt	3	4	69	69
Sumatran orangutan	Munich	0	5	22	22
Western lowland gorilla	Berlin	1	0	6	6
Western lowland gorilla	Frankfurt	4	6	83	83
Western lowland gorilla	Heidelberg	1	1	15	14
	Total Numbers	33	49	669	660

### Saliva Sampling Protocol

Saliva samples were collected throughout the day (07∶00 am –05∶00 pm). Apes had been trained to chew on cotton rolls which were soaked in a sugar solution in order to enhance their acceptance by the subjects [12]. Similar techniques have been used in previous studies (e.g. [31], [32], [33]) and some of these studies found evidence that cotton rolls that had been treated with a sugar solution may affect measurements of sAA (e.g. [31], [32]). Therefore, we extracted test rolls with deionized water (MilliQ®). There was no amylase reaction in test rolls or those collected from subjects. This suggests that preparation of cotton rolls did not cause cross-reactivity either in sAA or in the cortisol assay. For more information on cotton roll preparation and sampling procedure see Behringer et al. [12].

### Salivary Amylase and Saliva Cortisol Measurement

After collection, samples were stored at −20°C until analysis. After arrival in the lab, the thawed cotton rolls were centrifuged (1500 g, 10 min.) and sAA was measured in the endocrinology lab of the Max Planck Institute for Evolutionary Anthropology, Leipzig. Germany. For cortisol measurement, an aliquot of each sAA measured sample was shipped to the University of Veterinary Medicine, Vienna Department of Department of Biomedical Sciences, Vienna, Austria.

For cortisol measurements samples were diluted 1∶10 with assay buffer used for the cortisol assay. Using aliquots of the same saliva samples, cortisol was measured with an enzyme immunoassay (EIA) previously described by Palme & Möstl [34] and validated for apes by Behringer [35]. Intra- and inter-assay coefficients of the quality control in the cortisol assay were 11.6% (N = 28) and 12.3% (N = 74), respectively. Dilution of saliva samples for amylase depended on the species and individual levels and ranged from pure to 1∶50 (bonobos), 1∶10 to 1∶60 (gorillas), 1∶5 to 1∶300 (orangutans) and pure to 1∶20 (chimpanzees), respectively. Samples were diluted with buffer from the assay kit and 10 µl of the diluted saliva was applied to the assay. To measure sAA activity, we used the “Salivary alpha Amylase Enzymatic Assay” (RE80111, IBL International GmbH, Hamburg, Germany), a commercial enzymatic assay designed to measure alpha amylase activity in human saliva and validated for saliva of apes (for assay validation [12]). In brief, ten microliters of each pre-diluted standard, control, or sample was pipetted into each well and 200 µl of substrate solution was added per well with an 8-channel micropipette and incubated at room temperature (18–25°C). The first measurement was taken after 3 minutes incubation and a second one after 8 minutes of incubation. The intra- and inter-assay coefficients of variation of low and high value quality controls were 7.1% and 5.6% (N = 12) and 2.22% and 1.89% (N = 10), respectively. For both assays we re-assayed measurements if bindings were outside the linear range of the assay in an adequate dilution or if divergence of duplicates was greater than 10%. More information on sample preparation and assay operation procedures and recovery of sAA and cortisol is given in Behringer et al. [12].

### Statistical Analyses

To explore differences in sAA activity we used general linear mixed models (hereafter: GLMM, [36]). Models were run in R (R Development Core Team, 2011) using the function lmer provided by the package lme4 [37]. Models were fit with Gaussian error distribution and identity link function. For all models we tested various model diagnostics to assure that no assumption was violated (more details see below).

To investigate the influence of the predictor variables (a) species, (b) sex, (c) age and (d) sampling time (predictors with fixed effects) on sAA (log-transformed response variable) we used a GLMM into which we included, in addition to the main effects, all possible interactions between species, sex and age up to the three-way interaction. We also included subject identity and location (zoo) as random effects, to control for a possible influence of relevant animal husbandry conditions of each zoo such as diet, group size, and stress exposure. To achieve comparable estimates, time of sample collection and age of the animal were z-transformed to a mean of zero and a standard deviation of one [38]. An autocorrelation term was derived and integrated into the initial model. For this we used the same approach as described in Fuerthbauer et al. [39]. Since the autocorrelation term was negative and non-significant for sAA (EST. (estimate) = − 0.046, SE = 0.0316, t = 1.456) we removed it from the model.

The required normal distribution and homogeneity of residuals was tested by visual inspections of histograms, a qqplot of the residuals, and by plotting residuals against fitted values. Neither test indicated deviation from these assumptions. To establish the significance of the fixed effects as a whole we compared the full model with a null model excluding all fixed effects but retaining the random effects using a likelihood ratio test ([40]; R function “anova”). In order to achieve reliable P-values for the individual effects we used Markov chain Monte Carlo (MCMC) sampling to establish significance [36] using the functions pvals.fnc and aovlmer.fnc, respectively, as provided by the R package language [41]. To test for differences between sexes within species we built subsets for each of the four species. For every subset we ran a model with the sAA as response variable, sex, age and sampling time as fixed effects and zoo facility and subject as random effects. Additionally we built different subsets for males and females to test for differences between species. For each of the two subsets we ran a model with the sAA as response variable, species, age and sampling time as fixed effects and zoo and subject as random effects. We changed intercept position with the function “relevel” to assure that each species was compared to all others.

To investigate species and sex differences in salivary cortisol we included salivary cortisol (square root transformed) into a GLMM with the same fixed and random effects as described for sAA. Again, the autocorrelation term included was negative (EST = − 0.091, SE = 0.0260, t = − 3.512) and therefore removed from the model. As for sAA, visual inspection of plots did not indicate violation of the model assumptions.

We compared the full with the null model including the three-way interaction of sex, species and age with a likelihood ratio test ([40]; R function “anova”). As in the sAA model, MCMC was used with the functions pvals.fnc and aovlmer.fnc, respectively [41]. To test differences within species we used subsets of each species running separate models for each species with the salivary cortisol as response variable, sex, age and sampling time as fixed effects and zoo and subject as random effects. As in the case of sAA, we built subsets for each sex to compare species differences. We ran two models with salivary cortisol as a response variable, species, age and sampling time as fixed effects and zoo and subject as random effects. To rotate the intercept position we used the function “relevel”.

## Results

### Salivary Alpha Amylase Activity in Relation to Age, Sex, and Species

Comparing the full model with the null model and excluding all fixed effects but retaining the random effects revealed significant heterogeneity (χ^2^ = 109.32, df = 16, P<0.001). The initial model showed that the three-way interaction was not significant (P_MCMC = _0.1028) and therefore, it was removed from the model before running it again with all two-way interactions between the three fixed effects (species, sex, age). We found no significant interaction between species and age (P = 0.7436), nor between sex and age (P = 0.1758). However, the interaction between sex and species was significant (P = 0.0352). When removing the two non-significant interactions the P value remained significant (P = 0.0424). In the final model significant effects were found for age (P_MCMC_ = 0.0104) but not for time of sampling (P_MCMC_ = 0.3546).

#### Impact of sex, age, and sampling time

**Figure 1:**
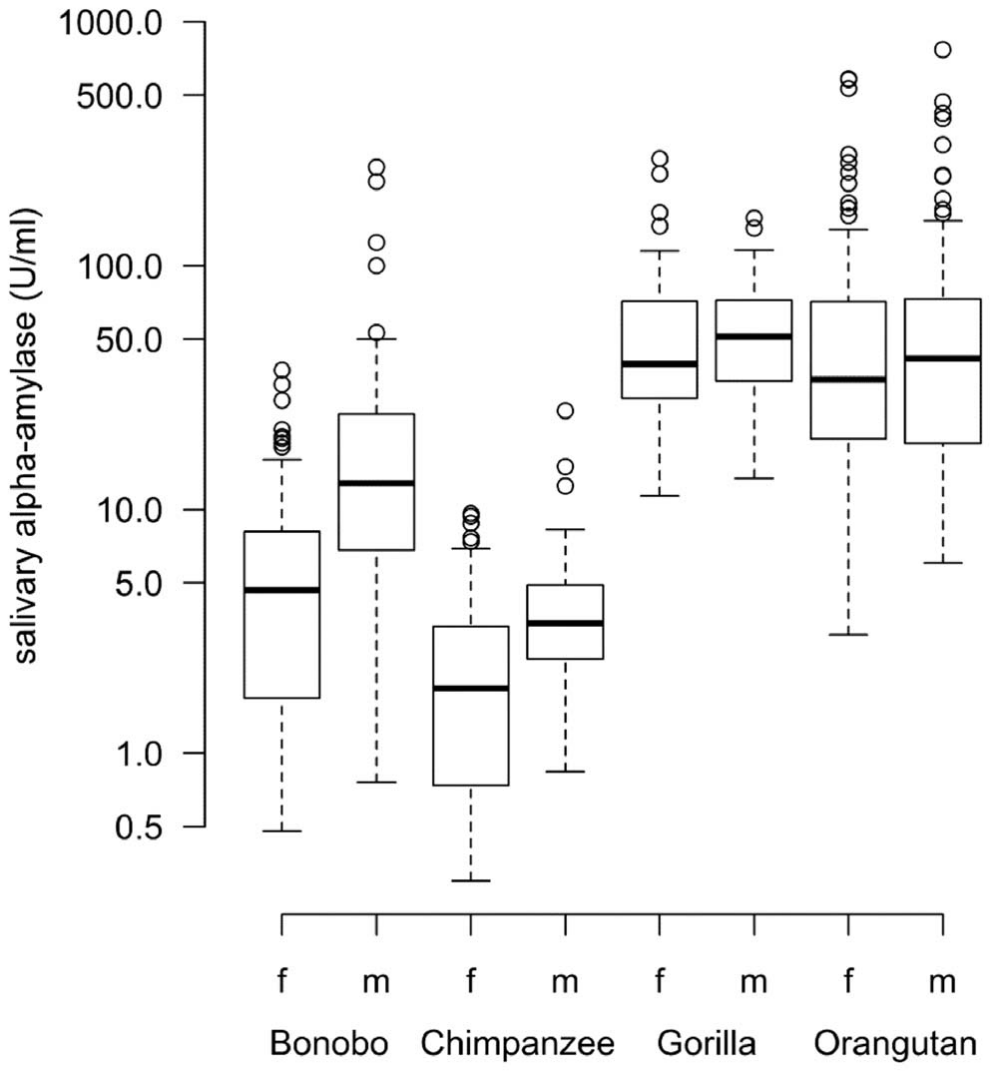
Average salivary alpha amylase (sAA) activity in females and males of the four ape species. The boxes illustrate the 25th and 75th percentiles, bars indicate medians, and circles indicate outliers. The y-axis is log transformed. Sample sizes: total N = 625: N_bonobo female_ = 92, N_bonobo male_ = 98; N_chimpanzee female_ = 113, N_chimpanzee male_ = 41; N_gorilla female_ = 60, N_gorilla male_ = 39; N_orangutan female_ = 107, N_orangutan male_ = 75.

Sex differences in sAA activity turned out to be significant for bonobos (P_MCMC_ <0.001) with males having higher sAA activity than females (Table 2 and Figure 1). In the other three species, females and males did not differ in their sAA activity (all P>0.05, for exact values Table 2). In terms of age, there was a significant increase of sAA with age in gorillas (Est. = 0.161, P = 0.046) and a trend for an increase of sAA with age in bonobos (Est. = 0.207, P = 0.079). There was no evidence that time of sampling had an impact on sAA activity (in all P>0.05, for exact values Table 2).

**Table 2:** Results of the general linear mixed models (GLMMs) of the four subsets for each species with sAA activity as response variable, and with age, time of day (both z-transformed) and sex as fixed effects.

	estimate	SE	P_MCMC_
**Chimpanzee**			
Intercept	0.841	0.395	0.084
sex	0.114	0.349	0.632
age	0.076	0.181	0.562
time of day	0.041	0.216	0.955
**Bonobo**			
Intercept	1.571	0.331	0.003
sex	1.043	0.336	**<0.001**
age	0.207	0.138	0.079
time of day	−0.024	0.087	0.765
**Gorilla**			
Intercept	3.789	0.134	0
sex	0.141	0.212	0.522
age	0.161	0.083	0.046
time of day	−0.080	0.069	0.302
**Orangutan**			
Intercept	3.683	0.383	0
sex	0.102	0.367	0.760
age	0.058	0.165	0.653
time of day	−0.155	0.148	0.281

The parameters zoo and subject were scored as random effects (MCMC = Markov Chain Monte Carlo; SE = Standard error).

#### Interspecies variation

In the female data set, sAA activity of bonobos and chimpanzees was found to be significantly lower compared to orangutans and gorillas, and compared to gorillas there was a trend for lower sAA activity in samples from orangutans (Table 3 and Figure 1). Males of both *Pan* species were also found to be significantly lower than gorillas and orangutans but males of the other two species did not differ in terms of sAA activity (Table 4 and Figure 1).

**Table 3:** Results of the general linear mixed model (GLMM) for subsets of females with sAA activity as response variable, species, age and time of day as fixed effects, and zoo and subject as random effects (MCMC = Markov Chain Monte Carlo; SE = Standard error).

		bonobo	chimpanzee	gorilla
chimpanzee	estimate	0.573	–	–
	SE	0.467	–	–
	P_MCMC_	**0.071**	–	–
gorilla	estimate	−2.375	−2.948	–
	SE	0.294	0.472	–
	P_MCMC_	**<0.001**	**<0.001**	–
orangutan	estimate	−1.83	−2.376	0.571
	SE	0.32	0.348	0.344
	P_MCMC_	**<0.001**	**<0.001**	**0.067**

**Table 4:** Results of the general linear mixed model (GLMM) for subsets of males with sAA activity as response variable, species, age and time of day as fixed effects, and zoo and subject as random effects (MCMC = Markov Chain Monte Carlo; SE = Standard error).

		bonobo	chimpanzee	gorilla
chimpanzee	estimate	1.586	–	–
	SE	0.383	–	–
	P_MCMC_	**<0.001**	–	–
gorilla	estimate	−1.359	−2.945	–
	SE	0.367	0.448	–
	P_MCMC_	**<0.001**	**<0.001**	–
orangutan	estimate	−1.287	−2.873	0.072
	SE	0.31	0.398	0.384
	P_MCMC_	**<0.001**	**<0.001**	0.752

### Salivary Cortisol Levels in Relation to Age, Sex, and Species

The full model was significantly different from the null model excluding the autocorrelation term and the three-way interaction of sex, age, and species (χ^2^ = 116.32, df = 16, P<0.001). As with sAA, the three-way interaction was not significant (P_MCMC = _0.1595) and was removed from the model. The model was run again with all two-way interactions and revealed no significant difference for the parameters sex and age (P = 0.159). For the interaction of species and sex, there was a trend (P = 0.065), and the interaction between species and age was significant (P = 0.0307). In the final model time of sampling was found to be highly significant (P_MCMC_ <0.001).

#### Impact of sex, age, and sampling time

**Figure 2:**
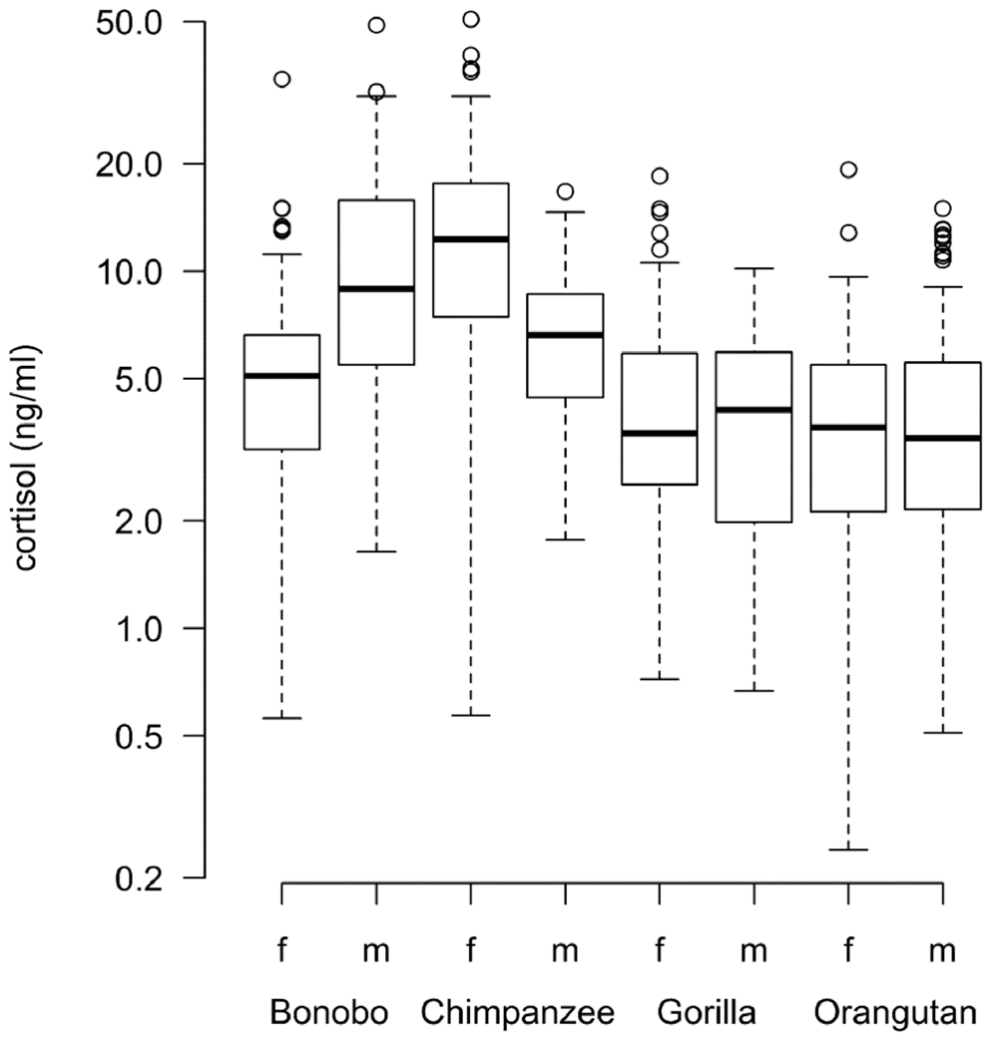
Average salivary cortisol concentrations for females and males of each species. The boxes illustrate the 25th and 75th percentiles, bars indicate median, and circles indicate outliers. Cortisol values on the y-axis are log transformed. Sample sizes: total N = 615, N_bonobo female_ = 90, N_bonobo male_ = 94; N_chimpanzee female_ = 112, N_chimpanzee male_ = 39; N_gorilla female_ = 59, N_gorilla male_ = 39; N_orangutan female_ = 107, N_orangutan male_ = 75.

To investigate the impact of sex, age, and sampling time, subsets of samples obtained from each species were used. Sex differences of cortisol values were found only in the samples from bonobos (P_MCMC_ = 0.0172) with males having higher salivary cortisol values than females (Table 5 and Figure 2). For the same species (but not the others), an age-related trend was found (P_MCMC_ = 0.063) with higher cortisol levels in samples from older individuals. Time of sampling was significant in all four species (P_MCMC_ <0.001) with higher cortisol values early in the morning followed by a decrease during the day.

**Table 5:** Results of the general linear mixed models (GLMMs) of the four subsets with salivary cortisol levels as response variable, and with age, time of day (both z-transformed) and sex as fixed effects.

	estimate	SE	P_MCMC_
**Chimpanzee**			
Intercept	2.374	2.374	0
sex	−0.047	0.404	0.686
age	−0.277	0.209	0.166
time of day	−1.239	0.249	**<0.001**
**Bonobo**			
Intercept	2.731	0.324	0
sex	0.581	0.275	**0.017**
age	0.179	0.109	**0.063**
time of day	−0.404	0.079	**<0.001**
**Gorilla**			
Intercept	2.249	0.209	0
sex	−0.153	0.233	0.397
age	−0.116	0.088	0.118
time of day	−0.306	0.068	**<0.001**
**Orangutan**			
Intercept	1.964	0.171	0
sex	0.087	0.129	0.429
age	−0.035	0.058	0.554
time of day	−0.448	0.079	**<0.001**

The parameters zoo and subject were scored as random effects (MCMC = Markov Chain Monte Carlo; SE = Standard error).

#### Interspecies differences

Salivary cortisol levels in samples from females of the two *Pan* species were significantly higher than in samples from gorillas and orangutans (Table 6 and Figure 2). In the subset of male samples, male bonobos had significantly higher salivary cortisol levels than males of the other three species (Table 7 and Figure 2).

**Table 6:** Results of the general linear mixed model (GLMM) obtained by analysing subsets of samples from females, with salivary cortisol concentration as response variable, with species, age and time of day as fixed effects, and zoo and subject as random effects (MCMC = Markov Chain Monte Carlo; SE = Standard error).

		bonobo	chimpanzee	gorilla
chimpanzee	estimate	−0.235	–	–
	SE	0.346	–	–
	P_MCMC_	0.442	–	–
gorilla	estimate	0.391	0.626	–
	SE	0.238	0.346	–
	P_MCMC_	**0.044**	**0.043**	–
orangutan	estimate	0.529	0.764	0.138
	SE	0.249	0.263	0.264
	P_MCMC_	**0.011**	**<0.001**	0.569

**Table 7:** Results of the general linear mixed model (GLMM) obtained by analysing subsets of samples males, with salivary cortisol concentration as response variable, with species, age and time of day as fixed effects, and zoo and subject as random effects (MCMC = Markov Chain Monte Carlo; SE = Standard error).

		bonobo	chimpanzee	gorilla
chimpanzee	estimate	0.807	–	–
	SE	0.377	–	–
	P_MCMC_	**0.008**	–	–
gorilla	estimate	1.02	0.223	–
	SE	0.288	0.384	–
	P_MCMC_	**0.001**	0.439	–
orangutan	estimate	1.058	0.251	0.023
	SE	0.246	0.33	0.299
	P_MCMC_	**<0.001**	0.34	0.952

## Discussion

In a previous study, we found that variation in sAA activity in bonobos showed sex differences and that sAA levels increased in stressful situations [12]. The results of the current study revealed that such sex differences in sAA activity are not a common trait of hominoid primates and that sAA activity differs across species. Basal sAA activity was higher in gorillas and orangutans than in the two *Pan* species. Within *Pan*, females did not differ in their sAA activity while male bonobos as a group had a significantly higher value than any other group of the *Pan* data set. Measures of salivary cortisol concentration showed that gorillas and orangutans had lower values compared to *Pan*. The highest cortisol concentrations were found in saliva samples from male bonobos. In addition to that, within-species variation in sAA activity was found to be related to age (e.g. gorilla). In humans differences in sAA activity reflect population-specific differences in AMY1 copy numbers [19] and the same relationship has been proposed to exist in non-human primates [17]. Although high numbers of AMY1 copies are usually related to the intake of dietary starch [42]. Animal studies found that sAA has an affinity to bind tannin, suggesting that high levels of sAA activity may have evolved in species (or populations) consuming a diet rich in tannins [43]. While it seems reasonable to infer that the variation in sAA between the different species of hominoid primates found in this study may also be related to dietary patterns and the corresponding genetic disposition (AMY1 copy number), it remains to be seen which nutritional component drives the inter-species differences in sAA in the four species.

### Between-species Differences in sAA Activity and AMY1 Copy Number

Assessment of AMY1 copy numbers in hominoid primates is beyond the scope of our study and we will relate our results on sAA activity to published information from genetic studies. There are two genetic loci for amylase, AMY1 (salivary amylase) and AMY2 (pancreatic amylase). AMY1 is found in human and non-human primates [44] and the African hominoids are known to differ in terms of AMY1 with gorillas having a higher number of copies compared to chimpanzees [16], [24] whereas bonobos have at least four AMY1 diploid copy numbers [16]. Keeping in mind the lack of information on AMY1 copy numbers in orangutans, the species differences in sAA activity found in our study correspond with information on AMY1 copy numbers. Based on the high sAA activity one would expect orangutans of having larger numbers of AMY1 copies than *Pan*.

### Between-species Differences in sAA Activity and Diet

The genetic disposition for a high sAA activity is considered to reflect an adaptation to the increasing significance of starch in the diet of early humans (e.g. [42]) and part of the AMY1 polymorphism found in modern humans is also explained by differences in dietary patterns. For example, Perry et al. [16] found a positive relation between number of AMY1 copies and dietary starch. The ape species involved in our study consumed a similar diet which consisted of a mix of fruits and vegetables supplemented with small amounts of cereals and, occasionally, animal protein. Although we did not analyse all of the food items provisioned during this study, nutritional analyses of the foods provisioned to apes of the same zoo facility did not indicate between-species differences in terms of dietary starch and/or tannin (own unpublished data). Adaptations in terms of digestive kinetics and digestive efficiency may be fairly resistant and persist when subjects are exposed to artificial diets. For example in a study on captive primates it was found that mean ingesta retention time reflects the characteristic patterns of the natural diet [45]. Milton [46] provisioned two species of New World monkeys that differ in terms of natural diet and gut morphology with identical food items and found that the two species maintained the species-specific patterns of passage rate. Assuming that the differences in sAA activity found in our study reflect adaptations to the natural diet, information from field studies are needed to explain the observed differences in sAA activity. Detailed information on diet composition of African apes is available from multiple sites (see publications in [47] and references therein). Although habitats vary in terms of nutritional ecology and although populations vary in terms of the type of plant foods they exploit [48], [49], the diet of bonobos and chimpanzees is similar in terms of the intake of nutrients and anti-feedants [49]. Chimpanzees prefer fruit which are high in sugar and low in tannin [50] and consumers maintain relatively high intake of fruit even at times of fruit scarcity [51]. While the diet of bonobos is also dominated by fruits, the species also consumes larger amounts of terrestrial and aquatic herbs [52]. Comparative analyses of the nutritional content of plant foods consumed by the two *Pan* species revealed that chimpanzees focus on high energy sources such as fat, while bonobos have higher intakes of protein and non-structural carbohydrates including starch [47]. However, inclusion of information from other chimpanzee populations did not confirm this pattern making the intake of starch by the two *Pan* species more similar [49].

Gorillas occupy different types of forest habitats and their diet varies considerably (e.g. [53], [54], [55]). Intake of fruit is seasonal in Western gorillas and absent in some populations of mountain gorillas and vegetative plant parts such as leaves, herbs, and bark account for a large proportion of their diet [56]. Compared to *Pan*, the diet of gorillas is high in structural carbohydrates and is assumed to contain relatively large amounts of tannins [56], [57]. In some populations, gorillas consume roots of various plant species that may not be eaten by sympatric chimpanzees [47], [58] which may lead to a relatively high intake of dietary starch.

Orangutans are highly frugivorous [59], [60], [61]. However, unlike populations living on the island of Sumatra, the diet of Borneo orangutans is constrained by seasonality in fruit abundance [60]. Periods of fruit scarcity may account for 4–5 months per year [60] and at such times consumers exploit sources of nutritionally poor plant foods such as cambium which is likely to increase the intake of both starch and tannins [59], [60]. Given the low nutritional value of cambium, it is reasonable to assume that the ability to extract digestible starch may be critical for populations that are exposed to pronounced fluctuations in high quality plant foods. Furthermore, orangutans are known as seed predators [59], [62]. Seeds are likely to be rich sources of starch and, at the same time, may contain high levels of anti-feedants such as tannins and both factors may promote high levels of sAA activity in orangutans.

While the descriptive accounts on dietary patterns appear to be in line with the species-differences in sAA activity of the four ape species, information from published reports on diet composition alone does not allow to draw major conclusions concerning species-differences in the consumption of starch or the load of tannins in the diet of wild apes and the topic requires more detailed investigation. One key to understand between-species variation in sAA activity is to compare the content of starch in different food categories. For example, it has been suggested that plants consumed at times when preferred food items are scarce contain more starch and that high levels of sAA may be adaptive in making efficient use of such fallback foods [25].

### Between-species Differences in sAA Activity and Stress

The results obtained in this study are in line with previous findings showing exceptionally high sAA activity only in male bonobos [12]. Males of this species also had the highest concentration of salivary cortisol which supports the idea that the high sAA activity is indicative of stress. In an experimental study it was found that bonobos are highly responsive to stress and show increasing salivary cortisol even when stress is anticipated [49]. Unlike chimpanzees, adult bonobos are co-dominant it has been reported that and females form alliances against males [63]. In captivity, males can be exposed to severe aggression from females and in some cases, joint female attacks may have fatal consequences for the male victim [35]. Low predictability of aggression could lead to uncertainty about the prospect of receiving aggression and produce chronic levels of stress [64]. Under natural conditions, communal aggression against males seems to be rare but there is anecdotal evidence that males may also be exposed to intense aggression [65]. While direct comparison of cortisol measurements is difficult, the combined evidence from behavioural and hormonal studies suggests that male bonobos may have to cope with high levels of stress.

Information obtained from samples of the three other hominoid species suggests within-species consistency in terms of sAA activity and salivary cortisol concentration. The finding that females of both *Pan* species had lower sAA activity but higher salivary cortisol concentrations than females of the two other species suggests that females differ in terms of their response to stress. Possible mechanisms affecting the response to stress are diverse and include receptor sensitivity and behavioural coping mechanisms [66]. However, more detailed studies are required to assess the impact of stress on the behaviour and physiology of hominoid primates and experimental approaches may help to understand how the different social systems of hominoids determine the response to stress and the mechanisms to cope with temporary elevated stress levels.

### Conclusion

Given the differences in sAA activity reported here, future field studies on hominoid primates may focus attention on specific dietary components such as digestible starches and tannin. In addition, the information from our study may guide molecular studies on AMY1 copy numbers in species such as orangutan and chimpanzee where populations occupy different habitats and are exposed to site specific environmental constrains. While the combined information on sAA activity and AMY1 copy numbers is useful for interpreting variation in diet within and across species, the impact of pancreatic alpha amylase activity is essential for the capacity to digest starches and needs to be taken into account before making inferences on the interaction between diet, genetic constitution, and enzymatic activity.

## Supporting Information

Table S1 **Age of apes at sampling time sorted in alphabetical order by species, location, sex and name.**
(DOC)
