# Supplementary material for: Measurements of Salivary Alpha Amylase and Salivary Cortisol in Hominoid Primates Reveal Within-Species Consistency and Between-Species Differences
Source: PLoS One. 2013 Apr 17;8(4):e60773. doi: 10.1371/journal.pone.0060773 (PMC3629192; doi:10.1371/journal.pone.0060773)
Supplement: Table S1 — Age of apes at sampling time sorted in alphabetical order by species, location, sex and name. (DOC) [file pone.0060773.s001.doc]

Table S1: Age of apes at sampling time sorted in alphabetical order by species, location, sex and name.

| species | location | sex | name | age sampled |
| --- | --- | --- | --- | --- |
| bonobo | Berlin | m | Kivu | 4 |
| bonobo | Berlin | m | Limbuko | 16 |
| bonobo | Berlin | m | Santi | 30 |
| bonobo | Frankfurt | m | Heri | 9 |
| bonobo | Frankfurt | m | Kelele | 6 |
| bonobo | Frankfurt | m | Ludwig | 26 |
| bonobo | Frankfurt | m | Nyota | 1 |
| bonobo | Frankfurt | f | Haiba | 7 |
| bonobo | Frankfurt | f | Kamiti | 21 |
| bonobo | Frankfurt | f | Kutu | 12 |
| bonobo | Frankfurt | f | Magrit | 49 |
| bonobo | Frankfurt | f | Nakala | 3 |
| bonobo | Frankfurt | f | Natalie | 42 |
| bonobo | Frankfurt | f | Omanga | 2 |
| bonobo | Frankfurt | f | Pangi | 1 |
| bonobo | Frankfurt | f | Ukela | 25 |
| bonobo | Frankfurt | f | Zomi | 12 |
| bonobo | Leipzig | m | Jasongo | 21 |
| bonobo | Leipzig | m | Joey | 29 |
| bonobo | Leipzig | m | Kuno | 15 |
| bonobo | Wuppertal | m | Lusambo | 31 |
| chimpanzee | Berlin | m | Kalle | 29 |
| chimpanzee | Berlin | m | Pedro | 29 |
| chimpanzee | Berlin | f | Gusta | 31 |
| chimpanzee | Berlin | f | Soko | 21 |
| chimpanzee | Leipzig | m | Alex | 10 |
| chimpanzee | Leipzig | f | Alexandra | 11 |
| chimpanzee | Leipzig | f | Annet | 11 |
| chimpanzee | Leipzig | f | Fifi | 17 |
| chimpanzee | Leipzig | f | Fraukje | 34 |
| chimpanzee | Leipzig | f | Gertruida | 17 |
| chimpanzee | Leipzig | f | Natascha | 30 |
| chimpanzee | Leipzig | f | Pia | 11 |
| chimpanzee | Leipzig | f | Riet | 33 |
| chimpanzee | Leipzig | f | Sandra | 17 |
| chimpanzee | Leipzig | f | Tai | 8 |
| chimpanzee | Munich | m | Toni | 50 |
| chimpanzee | Munich | m | Walter | 8 |
| chimpanzee | Munich | m | Willi | 12 |
| chimpanzee | Munich | f | Hannerl | 18 |
| chimpanzee | Munich | f | Püppi | 18 |
| chimpanzee | Nordhorn | m | Sunny | 29 |
| chimpanzee | Nordhorn | f | Biene | 27 |
| chimpanzee | Nordhorn | f | Lomela | 22 |
| chimpanzee | Nordhorn | f | Nancy | 32 |
| gorilla | Berlin | m | Ivo | 23 |
| gorilla | Frankfurt | m | Kabuli | 3 |
| gorilla | Frankfurt | m | Matze | 50 |
| gorilla | Frankfurt | m | Nasibu | 1 |
| gorilla | Frankfurt | m | Viatu | 13 |
| gorilla | Frankfurt | f | Dian | 19 |
| gorilla | Frankfurt | f | Fossey | 8 |
| gorilla | Frankfurt | f | Julchen | 43 |
| gorilla | Frankfurt | f | Makulla | 51 |
| gorilla | Frankfurt | f | Rebecca | 25 |
| gorilla | Frankfurt | f | Ruby | 11 |
| gorilla | Heidelberg | m | Bobo | 19 |
| gorilla | Heidelberg | f | Kiki | 4 |
| orangutan | Berlin | m | Bagus | 9 |
| orangutan | Berlin | m | Enchen | 22 |
| orangutan | Berlin | m | Kevin | 29 |
| orangutan | Berlin | m | Mano | 34 |
| orangutan | Berlin | f | Bini | 31 |
| orangutan | Berlin | f | Djasinga | 8 |
| orangutan | Berlin | f | Mücke | 21 |
| orangutan | Berlin | f | Satu | 5 |
| orangutan | Frankfurt | m | Charly | 51 |
| orangutan | Frankfurt | m | Galdikas | 6 |
| orangutan | Frankfurt | m | Lucu | 2 |
| orangutan | Frankfurt | f | Djambi | 48 |
| orangutan | Frankfurt | f | Jahe | 4 |
| orangutan | Frankfurt | f | Rosa | 19 |
| orangutan | Frankfurt | f | Sirih | 15 |
| orangutan | Krefeld | m | Barito | 11 |
| orangutan | Krefeld | m | Telok | 48 |
| orangutan | Krefeld | f | Lea | 18 |
| orangutan | Krefeld | f | Sungai | 7 |
| orangutan | Munich | f | Isalie | 3 |
| orangutan | Munich | f | Jolie | 2 |
| orangutan | Munich | f | Jula | 9 |
| orangutan | Munich | f | Liah | 8 |
| orangutan | Munich | f | Sitti | 22 |
